# Correction to: ‘Bayesian inference on a microstructural, hyperelastic model of tendon deformation’ (2022) by Forsyth *et* *al*.

**DOI:** 10.1098/rsif.2023.0554

**Published:** 2023-10-11

**Authors:** Jessica E. Forsyth, James Casey, Simon L. Cotter, William J. Parnell, Tom Shearer

**Keywords:** tendon, modelling, microstructural, hyperelastic, Bayesian, uncertainty

*J. R. Soc. Interface* **19**, 20220031 (Published online 18 May 2022). (https://doi.org/10.1098/rsif.2022.0031)

There was an error in the implementation of eqn (5.2) of [1] in the R package included in the electronic supplementary material. When corrected, this led to the following amendments:

— The 95% credible intervals for *ϕE* for the CDET data using the ST model at the end of §5.2 and the GT model in paragraph 3 of §5.3 are now 1360–1380 MPa in both cases.
— Figures 5, 7, 9, 11, 12 and 13 have been updated below. Figures 6, 8 and 10 are also affected, theoretically, but the changes to those figures are imperceptible by visual inspection.
— We have updated the R package and figures 5–8 in the additional mathematical material in the electronic supplementary material.

**Figure 5.**
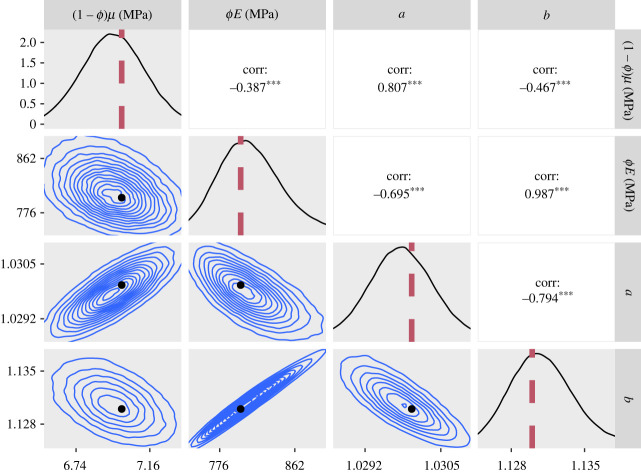
Plots of the posterior distributions calculated using the RWM algorithm on synthetic data. Main diagonal: marginal posteriors. Lower half: two-dimensional contour plots of the joint distributions. Upper half: posterior correlations between parameters. The parameter values used to create the synthetic data are represented by a red line on the posteriors and a black dot on the contour plots. For the correlation values, three asterisks represent *p* < 0.001. In order to create this figure, the 1 million samples were thinned by a factor of 10.

**Figure 7.**
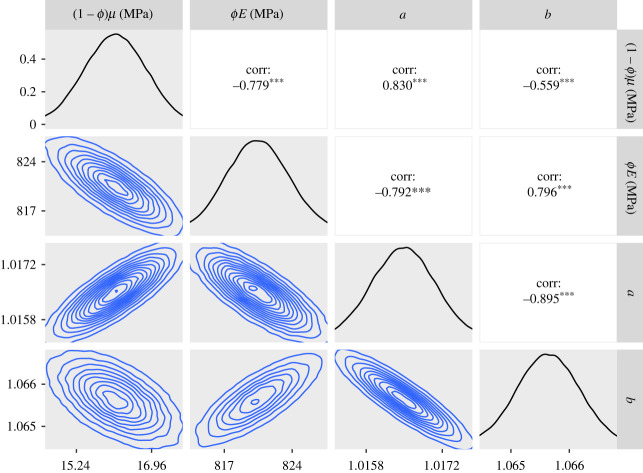
Approximate posteriors and contour plots of the parameters for the SDFT data. Samples were thinned by a factor of 10.

**Figure 9.**
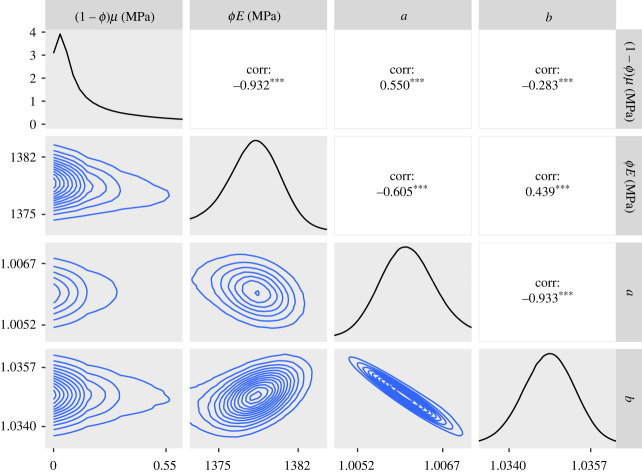
Approximate posteriors and contour plots of the parameters for the CDET data. Samples were thinned by a factor of 10.

**Figure 11.**
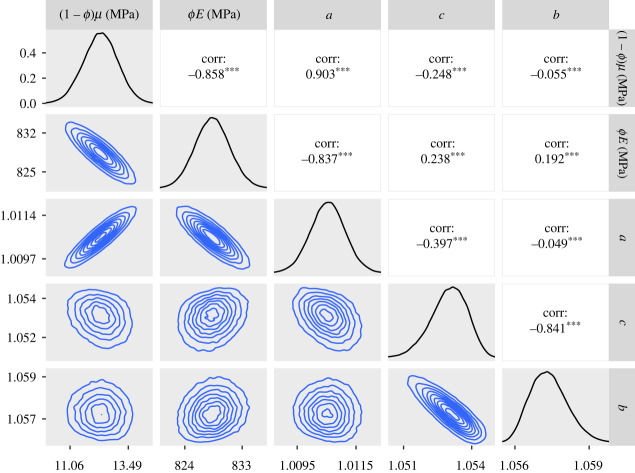
Approximate posteriors and contour plots of the parameters of the GT model for the SDFT data. Samples were thinned by a factor of 10.

**Figure 12.**
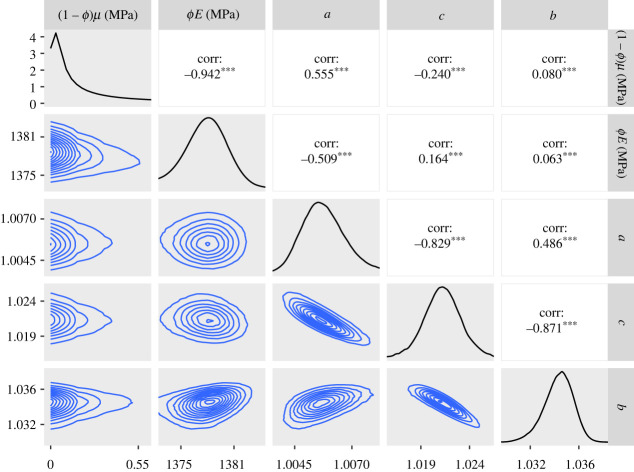
Approximate posteriors and contour plots of the parameters of the GT model for the CDET data. Samples were thinned by a factor of 10.

**Figure 13.**
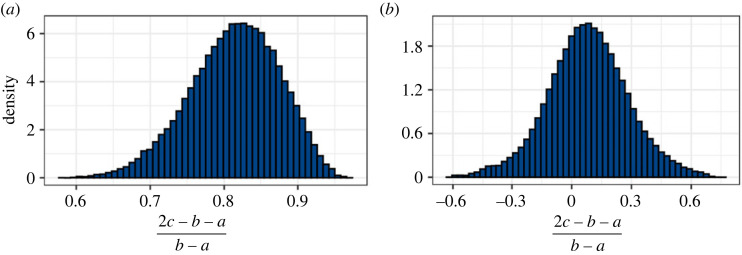
Histograms of (2*c* − *b* − *a*)/(*b* − *a*) for (*a*) the SDFT data and (*b*) the CDET data; (2*c* − *b* − *a*)/(*b* − *a*) ranges between −1 (*c* = *a*) and 1 (*c* = *b*) and 0 corresponds to an ST distribution.

All other results and the conclusions drawn are unaffected.

This has been corrected on the publisher's website.
